# *Salmonella* Typhimurium Infections Associated with a Community College Microbiology Laboratory — Maine, 2013

**Published:** 2013-11-01

**Authors:** Nicholas Matluk, Heather Grieser, Amy Robbins, Lauren B. Ball, Anne Redmond Sites, Stephen Sears, Kate Colby, Megan Kelly

**Affiliations:** Maine Dept of Health and Human Svcs; Maine Dept of Health and Human Svcs, Univ of Southern Maine

On May 2, 2013, a case of salmonellosis was reported to the Maine Center for Disease Control and Prevention. The patient reported symptoms of diarrhea, fever, abdominal pain, and nausea, after attending a community college microbiology laboratory class. A second case was reported on May 8. Epidemiologic interviews conducted with both patients indicated common exposure at a community college, including one patient specifically naming the other patient.

On May 15, the Health and Environmental Testing Laboratory (HETL) determined that the clinical *Salmonella* isolates from stool specimens provided by outside hospital laboratories from both patients were indistinguishable by pulsed field gel electrophoresis (PFGE) analysis from a specimen used by the students during the microbiology class. The clinical isolates and laboratory class isolate all had a PFGE pattern indistinguishable from that of bacteria isolated during a national *Salmonella* Typhimurium outbreak in 2010 that was associated with clinical and teaching microbiology laboratories (1). No cases were reported from Maine during the 2010 outbreak.

On May 28, two members of HETL visited the college to assess laboratory practices and discuss PFGE results. On May 31, a survey on laboratory practices was e-mailed to 106 students enrolled in the microbiology laboratory course and was completed by 14 students. The low response rate was attributed to graduation and classes out of session. According to the survey results, only four of the 14 students said they always wore gloves, six said they sometimes wore gloves, and two said they rarely wore gloves. However, when they did wear gloves, 10 students said they washed their hands with soap and water after taking off the gloves.

The results of the PFGE analysis, case interviews, e-mail survey, and site visit suggested that the college laboratory was the source of the exposure, but it was unclear whether the exposure was the result of direct handling of the *Salmonella* culture, a spill, or contaminated equipment. Using *Salmonella* at this teaching level is contrary to the biologic safety guidelines issued in 2012 by the American Society for Microbiology (2), which clearly state that before biosafety level 2 (BSL-2) work, students should be competent performing BSL-1 activities. The laboratory practices survey and site visit identified several potential contamination hazards. These findings were similar to those from the 2010 national outbreak investigation (1): equipment in disrepair, inconsistent use of personal protective equipment, breakdowns in hand hygiene, inappropriate storage and handling of laboratory coats, and use in the laboratory of personal items not dedicated to the laboratory, including cell phones. This outbreak highlights a need to reinforce the specific recommendations for improvement that arose from the 2010 outbreak when working with infectious material in teaching laboratories (1,3).
